# Hypolipidemic properties of *Chlorella pyrenoidosa* organic acids via AMPK/HMGCR/SREBP‐1c pathway in vivo

**DOI:** 10.1002/fsn3.2014

**Published:** 2020-12-11

**Authors:** Jie Chen, Shiyu Gong, Xuzhi Wan, Xiaoxiang Gao, Change Wang, Feng Zeng, Chao Zhao, Bin Liu, Ying Huang

**Affiliations:** ^1^ College of Food Science Fujian Agriculture and Forestry University Fuzhou China; ^2^ National Engineering Research Center of JUNCAO Technology Fujian Agriculture and Forestry University Fuzhou China; ^3^ Engineering Research Center of Fujian‐Taiwan Special Marine Food Processing and Nutrition Ministry of Education Fuzhou China; ^4^ Key Laboratory of Marine Biotechnology of Fujian Province Institute of Oceanology Fujian Agriculture and Forestry University Fuzhou China

**Keywords:** AMPK signaling pathway, *Chlorella pyrenoidosa*, ethanol extract, hypolipidemic, lipid metabolism

## Abstract

The aim of this study was to explore the effects and mechanisms of 95% ethanol extract of *Chlorella pyrenoidosa* (CPE95) on lipid metabolism in hyperlipidemic rats. For the sake of chemical composition analysis of CPE95, liquid chromatography–mass spectrometry (LC‐MS) was used for determination. After treatment with CPE95, serum high‐density lipoprotein cholesterol content of the hyperlipidemic rats was increased, while the contents of cholesterol, triglyceride, and low‐density lipoprotein cholesterol were decreased strikingly. Moreover, the result of histopathology analysis showed that the accumulation and fatty deformation of the livers were relieved. Real‐time quantitative PCR and Western blotting were used to determine the expression levels of lipid metabolism‐related genes. The gene expression level of adenosine 5’‐monophosphate‐activated protein kinase was descended, and expressions of sterol regulatory element‐binding protein‐1c, acetyl‐CoA carboxylase, and 3‐hydroxy‐3‐methylglutaryl coenzyme A reductase were all downregulated in the CPE95‐treated rats. It suggested that CPE95 may effectively improve the hyperlipidemia in rats and would be potential for functional food component to reduce blood lipid.

## Graphical abstract

The aim of this study was to investigate the effects and mechanism of 95% ethanol extract of Chlorella pyrenoidosa (CPE95) on lipid metabolism in high‐fat diet‐fed rats. Chemical compositions of CPE95 were determined by ultra‐high performance liquid chromatography–quadrupole/time‐of‐flight mass spectrometry (UPLC‐Q‐TOF‐MS/MS). The present results that Chlorella pyrenoidosa organic acids could improve lipid metabolism disorders by regulating AMPK/HMGCR/SREBP‐1c gene expressions.

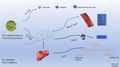

## 1 INTRODUCTION

The process by which lipids are synthesized, broken down, digested, absorbed, and transported is called lipid metabolism (Hou et al., 2019). Lipid metabolism disorder (LMD) usually leads to hyperlipemia, hypertension, cardiovascular diseases, obesity, and other diseases. Since such diseases account for more than 18 million deaths each year in the world, LMD has been recognized as a serious threat to global health (Fidèle et al., 2017). Experts predict that by 2020, the number of global lipid metabolism disorders will reach 78 million, including 13 million in China (Ni et al., 2015). Therefore, it is of great significance to balance lipid metabolism, and prevent and/or treat LMD for human beings. Although effective drugs against LMD have already been on the market for nearly half a century, the adverse effects of current drugs could not be negligible such as liver injury and gastrointestinal dysfunction (Licata et al., 2018). As a result, there is a rising requirement for novel drugs and/or nutraceuticals that could effectively prevent and/or treat LMD with low toxic and side effects. Emerging studies reported that some natural food nutrients or components could be potential for an effective treatment of LMD with higher safety (Al‐Gubory et al., 2016; Cao et al., 2014; Gómez‐Zorita et al., 2012; Zhang et al., 2014).

Well‐known for its high contents of nutritive substances, for example, polysaccharides, minerals, dietary fiber, protein, vitamins, and polyunsaturated fatty acids, and bioactive substances, *Chlorella* is a popular functional food material (Daugherty, 2014). At present, eicosapentaenoic acid (EPA) and docosahexaenoic acid (DHA) are a kind of preventive nutrients, which exist in *Chlorella pyrenoidosa* (Gómez‐Zorita et al., 2012; Zhang et al., 2014). The authority of the United Nations called *Chlorella* as "green health food" for its rich nutrition (Kotrbáček et al., 2015). In recent years, more and more pharmacological effects of *Chlorella* and its extracts have been reported, such as immune regulatory (An et al., 2016), antioxidant (Qi & Kim, 2018), anti‐inflammatory (Sibi & Rabina, 2016), anti‐allergic (Bae et al., 2013), and antitumor activities (Renju et al., 2014). In addition, *Chlorella* can not only regulate cholesterol synthesis and degradation, but also lower blood lipid level (Zhao et al., 2015; Noguchi et al., 2013). At present, a great deal of research has found that water extracts of *Chlorella* can regulate metabolic disorders (Wan et al., 2020) and that most of the water extracts are macromolecular active substances such as polysaccharides. It has been reported that organic acids, alkaloids, steroids, and other small molecular substances can regulate LMD (Li et al., 2019), which need to be extracted by organic solvent, are usually fat soluble. However, there is little information about the appraisal of the antilipidemic effect of the ethanol extract of *Chlorella*.

Complicated regulating network, involving the interaction of many signaling pathways, maintains the balance of metabolism. AMP‐activated protein kinase (AMPK) with its lower reaches, for instance, acetyl‐CoA carboxylase (ACC), 3‐hydroxy‐3‐methylglutaryl coenzyme A reductase (HMGCR), and sterol regulatory element‐binding protein‐1c (SREBP‐1c), has been reported as one of the important signaling pathways to regulate energy metabolism and lipid metabolism (Craig et al., 2018). It is necessary to explore its currently ambiguous mechanism of regulation of lipid metabolism, before the ethanol extract of *Chlorella* cloud be well applied to effective treatment of LMD. Therefore, the purpose of this study was to explore the effects and mechanisms of 95% ethanol extract of *Chlorella* (CPE95) on regulating lipid metabolism in hyperlipidemic rats.

## 2 MATERIALS AND METHODS

### 2.1 Preparation of CPE95

Dried *C. pyrenoidosa* powders were acquired by Fuqing King Dnarmsa Spirulina Co. Ltd. *C. pyrenoidosa* powder was mixed with 95% ethanol at a ratio of 1:10 (w/v) and placed in a 50 ℃ water bath for 1 hr. Then, the solution was subjected to centrifugation (5,000 rpm, 10 min), and the supernatant was concentrated by rotary evaporation and freeze‐drying. Ultimately, CPE95 was obtained and stored at −20°C.

### 2.2 Determination of the chemical composition of CPE95

The material composition of CPE95 was investigated by LC‐MS spectrometry analyzer (Shimadzu Corporation, Japan) with C18 column (1.8 μm, 2.1 × 100 mm) (Waters, Milford, MA, USA), based on previously published procedures (Huang et al., 2018). Briefly, the solvent A (0.1% (v/v) formic acid in water) and solvent B (acetonitrile) constituted the mobile phase, and the flow rate maintained at 0.3 ml/min. The gradient program is set according to the instructions: 5% B at 0–1 min, 5%–60% B at 1–7 min, 60%–80% B at 7–8 min, and 5% B at 8–10 min. The column was kept at 35 ℃, while the injection volume was 10 μL each time. The obtained eluent was directed to mass spectrometric analysis. The MS system uses electrospray ion source (ESI) and positive ionization mode. The scanning parameters are as follows: spray voltage 5,500 V, gas curtain gas stress 35 PSI, atomizing gas stress 55 PSI, assist gas stress 50 PSI, ion source temperature 500 ℃, solvent removal electric pressure 100 V, mass spectrum analysis 50–1700 *m/z*, and scanning speed 2 spectrum/s (Zhao et al., 2018). The data analysis was conducted by the software of masslynx 4.1 (Waters, Milford, Ma, USA).

### 2.3 Animals and CPE95 treatment

Thirty‐two male four‐week‐old Wistar rats (230 ± 10 g) were bought from Fuzhou Wu's Laboratory Animal Company and fed in the animal house of which temperature and relative humidity were maintained at 25℃ and 60%, respectively. And they were subjected to a 12:12‐hr dark photoperiod. During hebdomad of acclimation, the surviving rats were stochastically separated into 4 groups (*n* = 8 each) and provided with different feeding patterns during the 8 weeks of the experiment. Rats fed Lab Diet 5001 (Brentwood, Missouri, USA) were separate NFD group. The team in which rats were kept with high‐fat diet (HFD), which contains 67% NFD, 20% sugar, 10% lard, and 3% cholesterol, was named as the HFD group. The Evaluation of Chinese Health Foods recommended that the adult daily intake of *Chlorella* is 4–8 g, so we select 4 g and 8 g as the low dose and high dose, respectively. Besides, according to Shannon's study (Shannon et al., 2008), the dose conversion factor of adult (bw: 60kg) and mice (bw: 0.2 kg) is 6.25, so the gavage dose of rats is 150 mg/kg and 300 mg/kg. Besides being given food with HFD, the rats in the other two groups were also intragastrical administrated with 2 ml of CPE95 at two different doses (150 mg/kg·day, CPE95L) and (300 mg/kg·day, CPE95H) each day, respectively. By comparison, the rats within both NFD group and HFD group were rendered with 2 ml of 0.9% saline solution in the same manner. The weight of each group of rats was measured at the beginning, middle, and the end of experiment. The care and use of rats in this study were authorized by the Ethics Review Committee of the College of Food Science, Fujian Agriculture and Forestry University.

### 2.4 Sample collection

Before dissection, all rats were fasted for 12 hr to collect fecal samples and placed at 80°C. Then, all rats were euthanized in pentobarbital‐anesthetized condition (35 mg/kg). After dissection, blood samples of all rats were collected from the heart, could be put at 25°C for 2 hr, and next centrifuged (2,500 rpm, 10 min) to acquire serum samples, which was kept at 4°C. Liver sample of each rat was collected, weighed, and divided into two unequal parts. The smaller part was used for histopathological analysis. The rest was stored at −80°C for further use.

### 2.5 Histopathology analysis

In order to fix the sample, an appropriate amount of liver tissue was soaked in 4% paraformaldehyde, paraffin‐embedded, sliced, next stained with hematoxylin–eosin (H&E), and finally observed with 40 × optical microscope (SCOPE A1, ZEISS, Germany).

### 2.6 Biochemical analysis of serum samples

The levels of triglyceride (TG), total cholesterol (TC), high‐density lipoprotein cholesterol (HDL‐C), and low‐density lipoprotein cholesterol (LDL‐C) in rat serum were determined by the commercial kits (Jiancheng, Nanjing, China), including TG (A110‐1–1), TC (A111‐1), HDL‐C (A112‐1–1), and LDL‐C (A113‐1–1) kits.

### 2.7 Determination of the content of total bile acid in fecal samples

The fecal sample was dissolved in 0.9% saline at a proportion of 1:9 (w/v) by vortex, and next centrifuged at 3,500 rpm for 10 min at 4°C. The kit for determining the contents of total bile acid (TBA) in the supernatants was bought from Jiancheng Bioengineering Company.

### 2.8 Quantifying the transcription levels of lipid metabolism‐related genes

A kit for extracting total RNA from liver samples was acquired from Japan Takara Biological Company. cDNA was synthesized in PCR thermal cyclers (ProFlex™ PCR; Thermo Fisher Scientific, Singapore) by using a Prime Script™ RT Reagent Kit with gDNA Eraser gained from Takara Biological Company. Then, the transcription levels of some lipid metabolism‐concerned genes (comprising HMGCR, SREBP‐1c, ACC, and AMPK‐α genes) were quantified by real‐time quantitative PCR (RT‐qPCR) on ABI 7300 Real‐Time PCR System (ABI company, America) with the SYBR Premix Ex Taq II (Takara, Kusatsu, Japan). The primers for RT‐qPCR used in the experiment are shown in Table S1. And the PCR was conducted as below: denaturation at 95°C for 30 s, followed by 40 cycles of 95°C for 5 s, 60°C for 30 s, and 72°C for 30 s. mRNA expressions of genes were calculated using 2^−ΔΔCT^ method, and the mRNA expressions of genes in NFD group were set as *1* to calculate the relative mRNA expression of other groups. β‐actin was served as the reference gene.

### 2.9 Quantifying the gene expression levels by Western blot

A proper amount (0.1 g) of frozen rat liver was put into a 2‐ml centrifuge tube, and 0.9% normal saline was added in the proportion of 1:19 (w/v). Then, the liver sample was homogenized with the homogenizer (Hangzhou Miu Instruments CO.LTD, China) until it was completely dissolved, then placed in a centrifuge, adjusted the temperature to 4°C, the speed to 13200 ×*g*, and the time to 10 min, obtained the supernatant, and use BCA method to determine the protein content. Transferring an equal amount of 5 μL protein sample to a PVDF membrane (Millipore, USA) requires separation by SDS‐PAGE. After being blocked with QuickBlock™ Blocking Buffer (Takara, Dalian, China) at 4 ℃ for 40 min, the membrane was reacted with the primary rabbit polyclonal antibodies overnight at 4 ℃, and then second antibody combined with HRP‐labeled Goat Anti‐Rabbit IgG (H + L) (Beyotime Biotechnology, Shanghai, China) at 37 ℃ for 1.5 hr. Finally, the protein strips obtained were treated with the BeyoECL Moon Kit (Beyotime Biotechnology, Shanghai, China) and detected by GeneTools in syngene software of GeneGnome XRQ chemiluminescence imaging system (Synoptics Ltd, UK). GADPH was used as a drug loading control, and the protein expression levels of genes (gray value) were conducted by ImageJ software (1.52a; National Institutes of Health, USA). The value of the NFD group is the base number (set as *1*) to calculate protein expression levels in other groups.

### 2.10 Statistical analysis

The mean ± standard deviation (*SD*) represents the data of this experiment. SPSS 22.0 statistical software is used to perform one‐way analysis of variance (ANOVA) and Duncan's multiple comparison test. *p* < .05 was deemed as statistically marked difference, and *p* < .01 indicated extremely significant difference. The gray values of Western blot protein bands were converted into numerical values by Image J software (1.52a; National Institutes of Health, USA) for quantitative analysis. After obtaining the RT‐qPCR and Western blot data, the value of the NFD group was set as *1*, and relative levels of both mRNA and protein expression of the other groups were calculated against that of NFD group. Graphics were generated with GraphPad Prism Version 7.00.

## 3 RESULTS

### 3.1 The chemical compositions of CPE95

**FIGURE 1:**
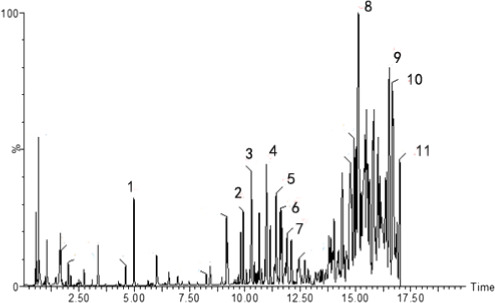
Chromatographic peak of CPE95 in UPLC

The chemical ingredient of CPE95 was determined by LC‐MS, and the peaks with ionic strength greater than 20% are selected. A total of 11 main peaks of CPE95 with the retention time ranging from 4.98 to 17.00 min were observed by UPLC (Figure 1). The information of each component obtained from the mass spectrometry results (Figure S1), such as *m/z* spectral data, time, parent peak, and fragment pattern, was compared with previously published documents. Hence, polyunsaturated fatty acids, alkaloids, flavonoid, and phenolic compound were identified in CPE95 (Table 1).

**Table 1:** Characterization of major components of CPE95 by UPLC/Q‐TOF‐MS/MS

No.	Rt (min)	Compound	Probable formula	Measured [M + H] ^+^ (*m/z*)	Representative reference fragmentation	References
1	4.98	Gluconic acid	C_6_H_12_O_7_	197.089	105.0406,107.0560,133.0710, 135.0866,166.0666,179.0775	Almalki et al., 2016
2	9.92	5,8,11,14‐Octadecatetraynoicacid		325.216	305.0974,304.0908,159.0867	
3	10.29	Lysophosphatidylcholine (18:3)	C_26_H_48_NO_7_P	518.3215	104.0778,124.9699,184.0442, 500.3077	Amessis‐Ouchemoukh et al., 2014
4	10.98	Lysophosphatidylcholine (18:2)	C_26_H_50_NO_7_P	520.337	104.0777,124.9696,184.0439, 502.3228	Choi et al., 2017
5	11.41	Lysophosphatidylcholine (16:0)	C_24_H_50_NO_7_P	496.3339	104.0777,124.9697,184.0440, 185.0474,478.3203	Sayed et al. 2016
6	11.61	Poricoic Acid A	C_31_H_46_O_5_	498.3732	236.1219,480.3600,144.0708	Li et al., 2018
7	11.91	Linoleic acid isomer	C_18_H_30_O_2_	337	335.1457,334.1399	Liang et al., 2018
8	15.12	Amurensin	C_26_H_29_O_12_	533.2509	370.0973,371.1169,489.1485, 504.2478,517.2534	Wan et al., 2018
9	16.52	Acetyl dicaffeoylquinic acid		559.4341	491.2531	
10	16.65	Galypumoside A	C_38_H_41_O_17_	769.5182	663.3832	Wu et al., 2018
11	17.00	Unknown		561.4514	493.2699,494.2737	

### 3.2 The effect of CPE95 on the body weights of HFD‐fed rats

**FIGURE 2:**
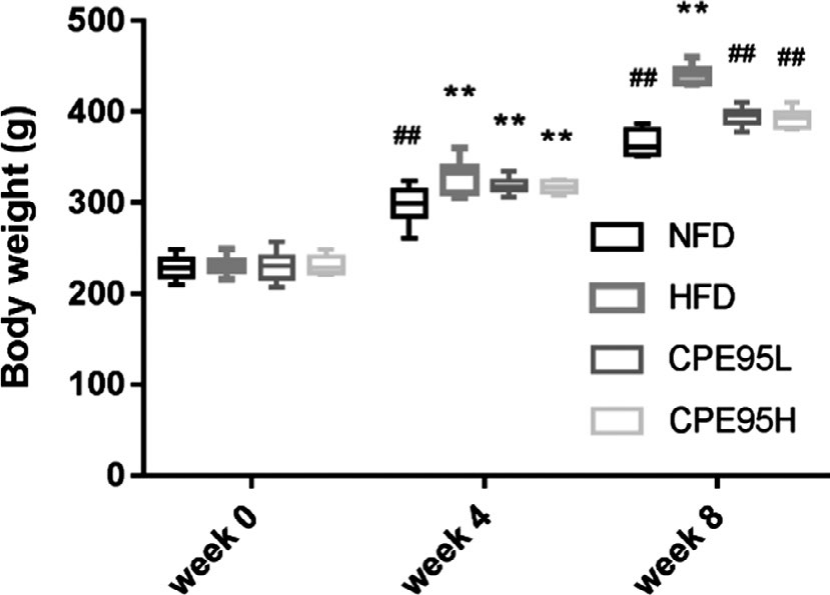
The weight changes of rats of different groups during the eight‐week experiment. All data are represented as the mean ± *SD* (*n* = 8). *p < .05 and **p < .01 versus NFD group; ^#^ *p* < .05 and ^##^ *p* < .01 versus HFD group

At week 0, there was little weight difference among four groups. After four weeks of the experiment, the weights of the rats in all groups increased; however, the weight was increasing at the lowest rate in NFD group. At week 8, the highest body weight among these four groups was the HFD group of rats, which was extremely significant from that of NFD, CPE95L, and CPE95H groups (*p* < .01). There was no significant difference in body weight between CPE95 groups (regardless of high or low dose) and NFD group (Figure 2). Therefore, our results suggested that CPE95 could alleviate body weight gaining in hyperlipidemia rats.

### 3.3 The effect of CPE95 on serum biochemical indexes of HFD‐fed rats

**FIGURE 3:**
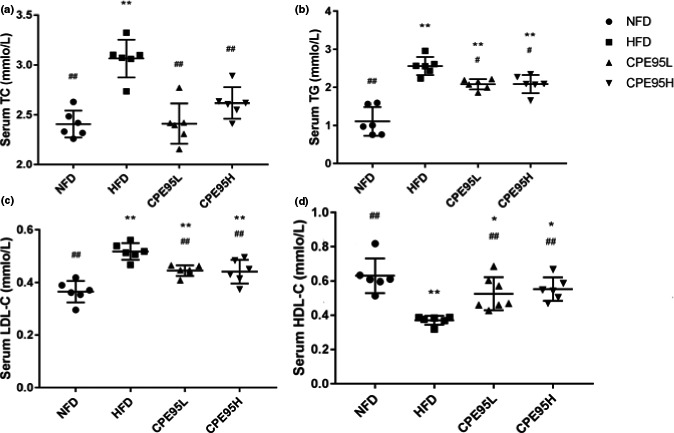
Effect of CPE95 on the contents of TC (a), TG (b), LDL‐C (c), and HDL‐C (d) of rats after 8‐week experiment. All data are represented as the mean ± *SD* (*n* = 8). *p < .05 and **p < .01 versus NFD group; ^#^ *p* < .05 and ^##^ *p* < .01 versus HFD group

The typical symptoms of LMD are increased standards of TC, TG and LDL‐C accompanied by decreased standard of HDL‐C, which can be seen in the HFD group of serum indicators (Figure 3). At week 8, the rats of HFD group had the highest value of TC, TG, and LDL‐C, but the lowest value of HDL‐C among four groups. Both CPE95 groups showed significantly lower TC content than HFD group (*p* < .01), and it should be noted that no difference of TC content was observed between NFD group and each CPE95 group. As for the experimental results of TG, LDL‐C, and HDL‐C, there were marked differences among each CPE95 group and HFD group (*p* < .05); also, striking differences were present among each CPE95 group and NFD group (*p* < .05). Therefore, experimental outcome showed that after CPE95 treatment, the contents of TC, TG, and LDL‐C of rats effectively decreased while HDL‐C content raised in HFD‐fed rats. Although they were still different from that of NFD group (except TC), it could be found that LMD caused by HFD was relieved with the application of CPE95.

### 3.4 The effect of CPE95 on liver tissue of HFD‐fed rats

**FIGURE 4:**
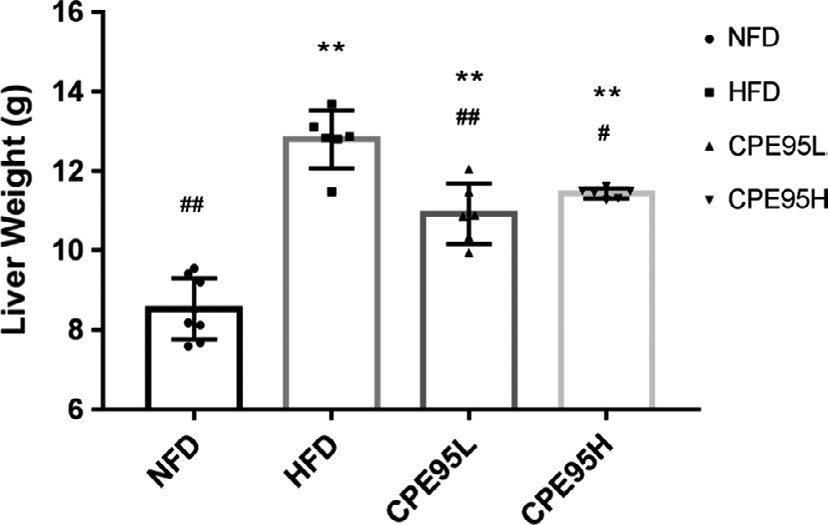
Liver weight of 4 groups of rats after 8 weeks of experiment. *p < .05 and **p < .01 versus NFD group; ^#^ *p* < .05 and ^##^ *p* < .01 versus HFD group

**FIGURE 5:**
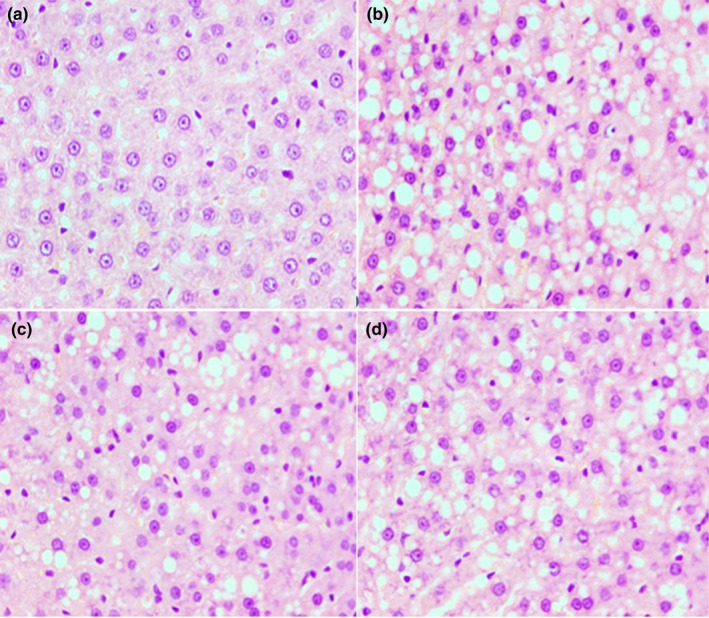
Histopathological analysis of the sections of rats after 8‐week experiment at 40×. NFD group (a), HFD group (b), CPE95L group (c), and CPE95H group (d)

HFD could lead to the weight gaining of the liver, which could be seen from the fact that the liver weights of normal rats group were obviously less than the other three groups (*p* < .01) (Figure 4). Liver weight heaviest belongs to the HFD group of rats. After treatment with CPE95, no matter high dose or low dose, the liver weights of rats were remarkable lower than HFD group (*p* < .05). Experimental outcomes showed that CPE95 had a certain defend effect on the liver tissue of rats against weight gaining. Histological structures of liver tissue sections from rats were observed, and the results are shown in Figure 5. In NFD group, hepatic cells showed complete morphology, clear nuclear, moderate size, and uniform distribution (Figure 5a). In comparison with NFD group, the liver sections of the HFD group of rats (Figure 5b) had obviously higher number and large shape of white lipid droplets, with the boundary of some hepatic cells being ambiguous, which indicated that the accumulation of liver fat increased and some hepatic cells degenerated. Due to the administration of CPE95, the morphology of hepatocytes in the CPE95L and CPE95H groups has been obviously improved (compared with the HFD group), which could be seen from the clearness of nuclear and a decrease in the number and the size of lipid droplets (Figure 5c‐d). This result indicates that fat accumulation in the liver was significantly reduced. Notably, the number and size of lipid droplets in the CPE95L group were smaller than those in the CPE95H group, which was consistent with the fact that the liver weight of the CPE95L group was lighter than that of the CPE95H group (Figure 4). The results suggested that CPE95 could improve lipid metabolism disorders and reduce liver lipid droplet deposition and liver weight at a 2 times lower dose (150 mg/kg·day compared to 300 mg/kg·day), which could possibly be related to the oral bioavailability. Similar results were reported by Du Preez (Preez et al., 2019). Therefore, the results of histopathology analysis suggested that CPE95 can mitigate hepatocyte injury induced by HFD.

### 3.5 The effect of CPE95 on fecal TBA of HFD‐fed rats

**FIGURE 6:**
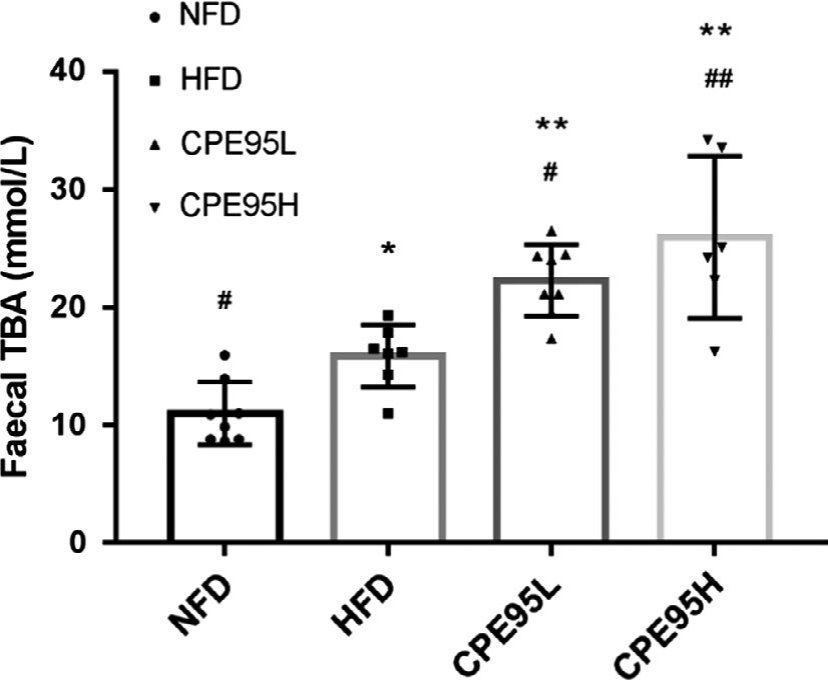
The total bile acid content in feces of 4 groups of rats after 8 weeks of experiment. *p < .05 and **p < .01 versus NFD group; ^#^ *p* < .05 and ^##^ *p* < .01 versus HFD group

The effect of CPE95 on fecal TBA is shown in Figure 6. After 8‐week feeding experiment, TBA contents in normal rats were lower than that in CPE95H group, CPE95L group, and HFD group (*p* < .05). Meanwhile, two CPE95 groups had significantly higher TBA levels than that of hyperlipidemia rats (*p* < .05). Moreover, it was observed that the rats had higher TBA content after treatment with higher dose of CPE95. These results suggested that CPE95 could lead to higher amount of TBA involving in the lipid metabolism.

### 3.6 The effect of CPE95 on gene transcription and expression in HFD‐fed rats

**FIGURE 7:**
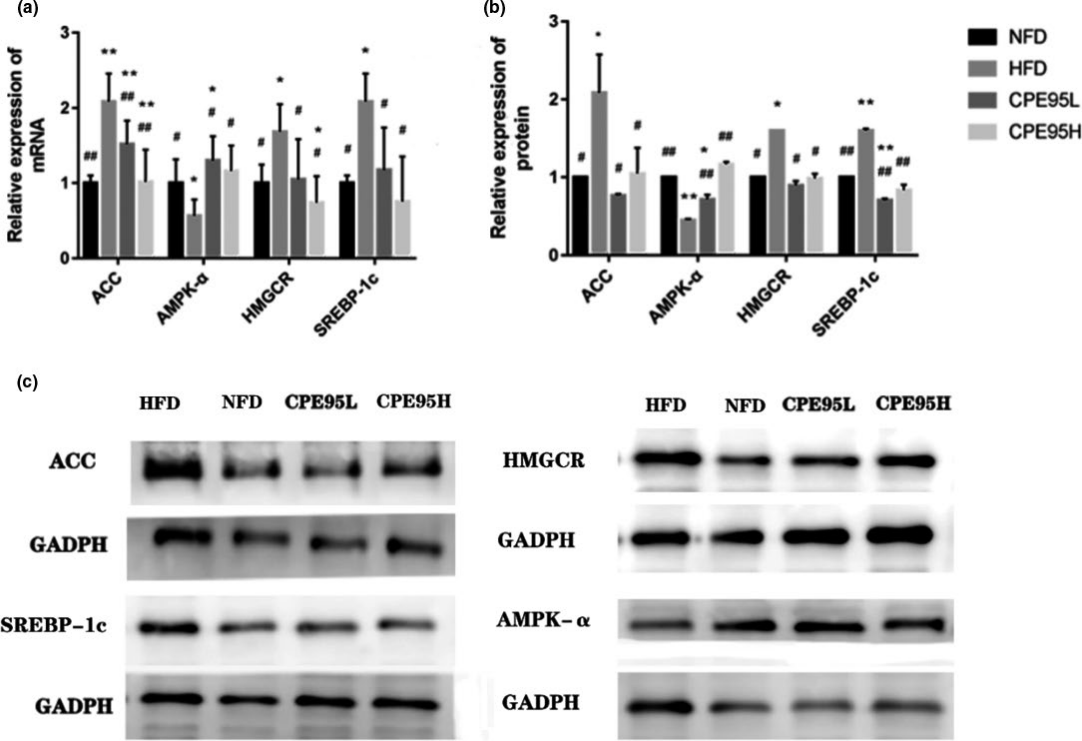
The effect of CPE95 on the expression of RNA and protein. of ACC, AMPK‐α, HMGCR and SREBP‐1c genes by RT‐qPCR and Western blot. *p < .05 and **p < .01 versus NFD group; ^#^ *p* < .05 and ^##^ *p* < .01 versus HFD group

In order to prove the potential molecular mechanism of CPE95‐regulating lipid metabolism, AMPK‐α, SREBP‐1c, ACC, and HMGCR transcription and expression levels, RT‐qPCR and Western blot experiments were used. As illustrated in Figure 7a, in comparison with HFD group, SREBP‐1c, ACC, and HMGCR gene mRNA transcription level in CPE95 groups were significantly downregulated, while AMPK‐α gene transcription was significantly upregulated. In addition, there was no striking difference in transcription levels of AMPK‐α and SREBP‐1c genes between CPE95H group and NFD group, and almost no striking difference in transcription level of HMGCR between CPE95L group and NFD group. As for the results of Western blot, the expression levels of AMPK‐α, SREBP‐1c, ACC, and HMGCR showed the same trend as the results of RT‐qPCR (Figure 7b‐7c). Therefore, CPE95 may play a lipid‐lowering effect by inhibiting the expression of genes related to fatty acid and cholesterol synthesis (Pan et al., 2019).

## 4 DISCUSSION

CPE95 was applied to the rats induced with HFD. Some biochemical indexes (serum TC, TG, LDL‐C, and HDL‐C), histopathology analysis of liver sections, plus expression standards of specific genes (AMPK‐α, SREBP‐1c, ACC, and HMGCR) were studied. After the supplement of CPE95, HDL‐C content in hyperlipidemia rats increased, while the numerical value of TC, TG, and LDL‐C descended observably, which was in accordance with early study (Miller et al., 2011). Emerging researches have indicated that serum TC, TG, HDL‐C, and LDL‐C are closely related to metabolic syndromes. LDL‐C is reported to be the main carrier of TC, which is regarded as an important index for the measurement of atherosclerotic cardiovascular disease, and increased levels of LDL‐C and TC may accelerate the atherosclerosis (Ai et al., 2018; Deedwania et al., 2016). CPE95 may promote the transport of TC and TG from adtevak to hepatic by raising serum HDL‐C standard and decreasing serum LDL‐C standard. In addition, we can clearly see that the liver tissues tended to be normal in hyperlipidemia rats with the intervention of CPE95, in terms of liver weights, the number and size of fat droplets, and hepatocyte morphology. Therefore, CPE95 showed good effect of improving lipid metabolism.

Bile acids play various physiological roles in the metabolism, which are considered to be metabolic integration factors and signaling molecules that regulate lipid homeostasis. In addition, bile acids can help digestion and absorption of esters, and are the ultimate products of cholesterol decomposition. In this study, due to higher intake of cholesterol, the content of TBA is different among the four groups of rats, and the content of normal rats is the lowest. However, the contents of TBA in the rats from CPE95 groups increased significantly than that of HFD group, which may be caused by the increase in cholesterol excretion or the inhibition of cholesterol synthesis after being treated with CPE95 (Pan et al., 2018). Such speculation was consistent with the fact that higher content of serum HDL‐C in both CPE95 groups than HFD group (Figure 3d). As we know, HDL‐C plays a vital part in the process of transporting cholesterol from peripheral tissues to liver, and cholesterol is further decomposed into bile acids. Similar result was also reported by Sano study, which found that *Chlorella* powder can significantly reduce cholesterol in rats fed with HFD by increasing fecal bile acid (Sano, 1982; Zhao et al., 2015). Moreover, the effect of CPE95 on TBA content was dose‐dependent, since the megadose group was found to have more remarkable effect than the low‐dose group. Therefore, the consequences indicated that CPE95 had a good regulatory impact on hyperlipidemic rats.

For the sake of further probe the mechanism of CPE95 regulating LMD, we studied the expressions of AMPK‐α, SREBP‐1c, ACC, and HMGCR genes participated in lipid metabolism. The results showed that CPE95 had a significant effect on the upregulation of AMPK‐α expressions and the downregulation of SREBP‐1c, ACC, and HMGCR. As an AMP‐activated protease, AMPK‐α is an enzyme necessary for keeping energy equilibrium and take an important role in adjusting lipid metabolism (Andris & Leo, 2015; Kohjima et al., 2008; Pearson et al., 2018). It may improve LMD by regulating expression of its lower courses genes, for instance, SREBP‐1c, ACC, and HMGCR (Chen et al., 2018). SREBP‐1c is one of the important genes regulating liver fat synthesis (Xu et al., 2001), which is in direct ratio to the extent of liver steatosis (Seo et al., 2009). AMPK‐α can restrain the expression of SREBP‐1c through phosphorylation of serine 372 and promote the protein hydrolysis process of SREBP‐1c, thus reducing lipid synthesis, eliminating lipid accumulation (Hardie, 2004), and inhibiting fatty degeneration (Suchankova et al., 2005; Wang et al., 2018; Xu et al., 2001). In terms of mRNA and protein expression (Figure 7), the expression level of SREBP‐1c in HFD group was striking higher than that in NFD group, thus stimulating fat production and lipid accumulation in vivo. ACC is an essential rate‐limiting enzyme in fatty acid metabolism, and also a key factor in regulating fatty acid synthesis and oxidation. AMPK‐α can inhibit the expression of ACC, while decreased expression of ACC can reduce the synthesis of glycogen, cholesterol, and protein, and inhibit the synthesis of fatty acids (Peng et al., 2019; Yang et al., 2010). In our study, it was obvious that high‐carbohydrate and high‐fat diet activated ACC expression (Figure 7). But the expression standard of ACC in CPE95 groups was notable less than that in HFD group, indicating that the expression of ACC gene was restrained after the intervention of CPE95. HMGCR, as a rate‐limiting enzyme in the biosynthesis process of cholesterol and other isoprene, catalyze the conversion of HMG‐CoA into methylvaleric acid. As a result, the inhibition of HMGCR can hinder cholesterol synthesis (Davies et al., 1990). It can be seen from our results (Figure 7) that HMGCR was downregulated in mRNA and protein expression in CPE95 groups, with significant difference compared with HFD group. The results of gene expression showed that CPE95 play a positive role in LMD via activating AMPK‐α and inhibiting the expression of HMGCR, SREBP‐1c, and ACC, which suggested that CPE95 has a positive effect on LMD.

According to previously reported MS fragmentation patterns, 10 compounds in CPE95 were characterized qualitatively in this study. The identified 10 compounds mainly belong to unsaturated fatty acids, alkaloids, and flavonoids, which have been demonstrated the regulation effect toward metabolic disorders, including hypolipidemic effect, in many studies (He et al., 2017; Li et al., 2019). Due to the fragmentation mode of the rest compound, peak no. 11 did not fit any published references we found, and further research is still needed to identify this eleventh compound in CPE95. Among 11 main compounds present in CPE95, whether all these compounds play a synergetic effect in lowering blood lipid, or the compounds which play the best role, could be clarified after further purification and characterization.

## 5 CONCLUSIONS

In summary, 95% ethanol extract of *C. pyrenoidosa* (CPE95) can significantly improve the blood lipid index and fecal bile acids of rats. After treated with CPE95, the liver weight of rat and the degree of liver degeneration were reduced, and the damage of liver cells was improved. At the same time, the genes referred to lipid synthesis and biodegrade were also adjusted by CPE95. The mRNA expression of AMPK‐α was increased, and the mRNA expressions of SREBP‐1c, HMGCR, and ACC were decreased, indicating that CPE95 has the latent to improve lipid metabolism chaos. Therefore, our consequences indicated that CPE95 likely be a desirable material to be applied in lipid‐lowering functional food.

## CONFLICT OF INTEREST

The authors declare no conflict of interest.

## ETHICAL APPROVAL

The research protocols were approved by the Fujian Agriculture and Forestry University (Approval No. FS‐2017–002). All procedures were conducted according to the relevant rules and regulations.

## Supporting information

Appendix S1
