# Supplementary material for: Hypolipidemic properties of Chlorella pyrenoidosa organic acids via AMPK/HMGCR/SREBP‐1c pathway in vivo
Source: Food Sci Nutr. 2020 Dec 11;9(1):459–68. doi: 10.1002/fsn3.2014 (PMC7802577; doi:10.1002/fsn3.2014)
Supplement: Supplementary file 1 — Appendix S1 [file FSN3-9-459-s001.docx]

| Gene | Forward primer | Reverse primer |
| --- | --- | --- |
| β-actin | 5′-GGAGATTACTGCCCTGGCTCCTA-3′ | 5′-GACTCATCGTACTCCTGCTTGCTG-3′ |
| ACC | 5′-ATGTGCCGAGGATTGATGG-3′ | 5′-TTGGTGCTTATATTGTGGATGG-3′ |
| AMPK-α | 5′-TCAGGCACCCTCATATAATC-3′ | 5′-TGACAATAGTCCACACCAGA-3′ |
| SREBP-1c | 5′-AAACCAGCCTCCCCAGAGA-3′ | 5′-CCAGTCCCCATCCACGAAGA-3′ |
| HMGCR | 5′-TGTGGGAACGGTGACACTTA-3′ | 5′-CTTCAAATTTTGGGCACTCA-3′ |

**Table S1.** List of primers used to amplify mRNA by RT-qPCR.


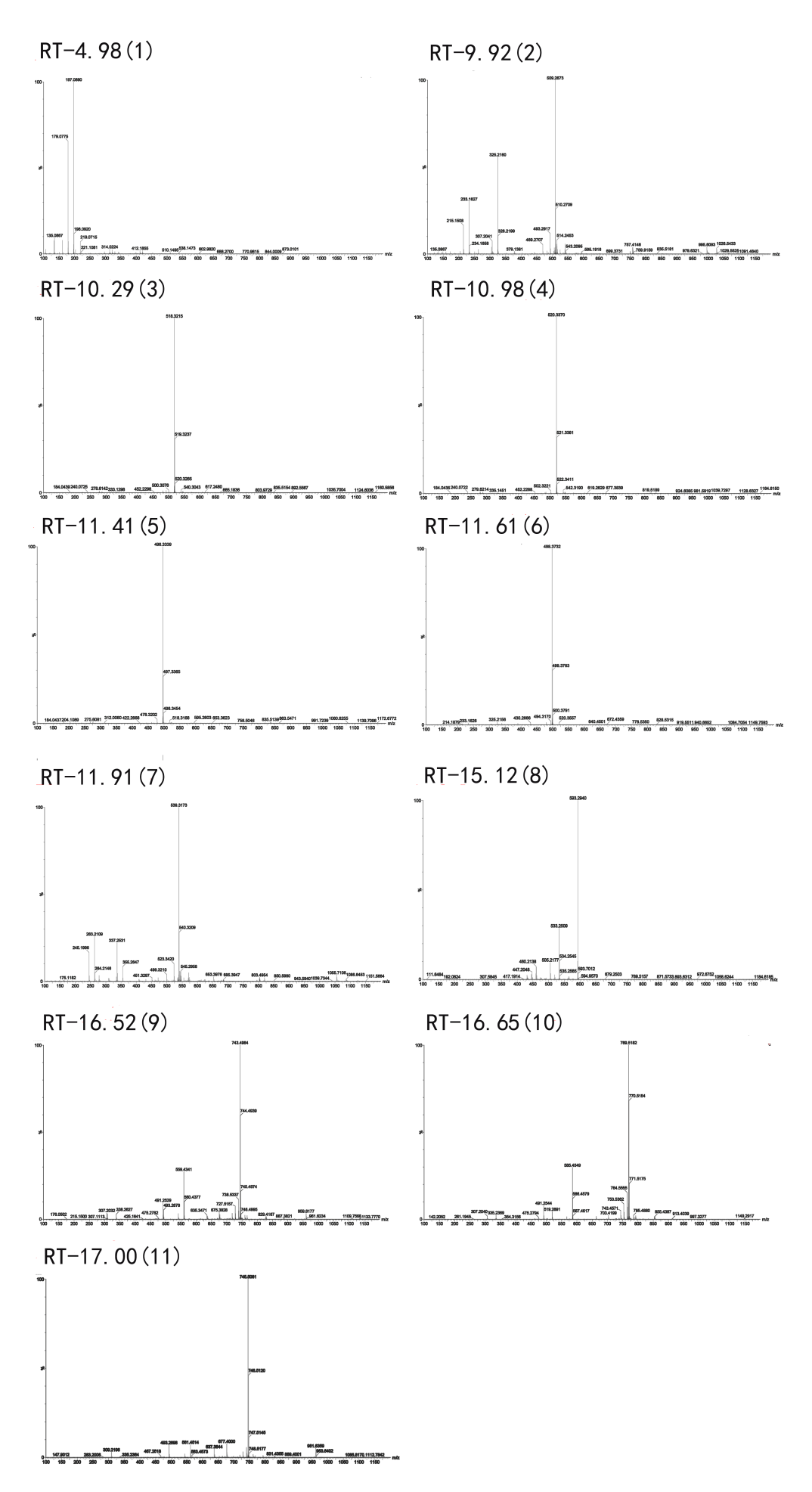


**Figure S1.** The main components of CPE95 identified by UPLC/Q-TOF-MS/MS.


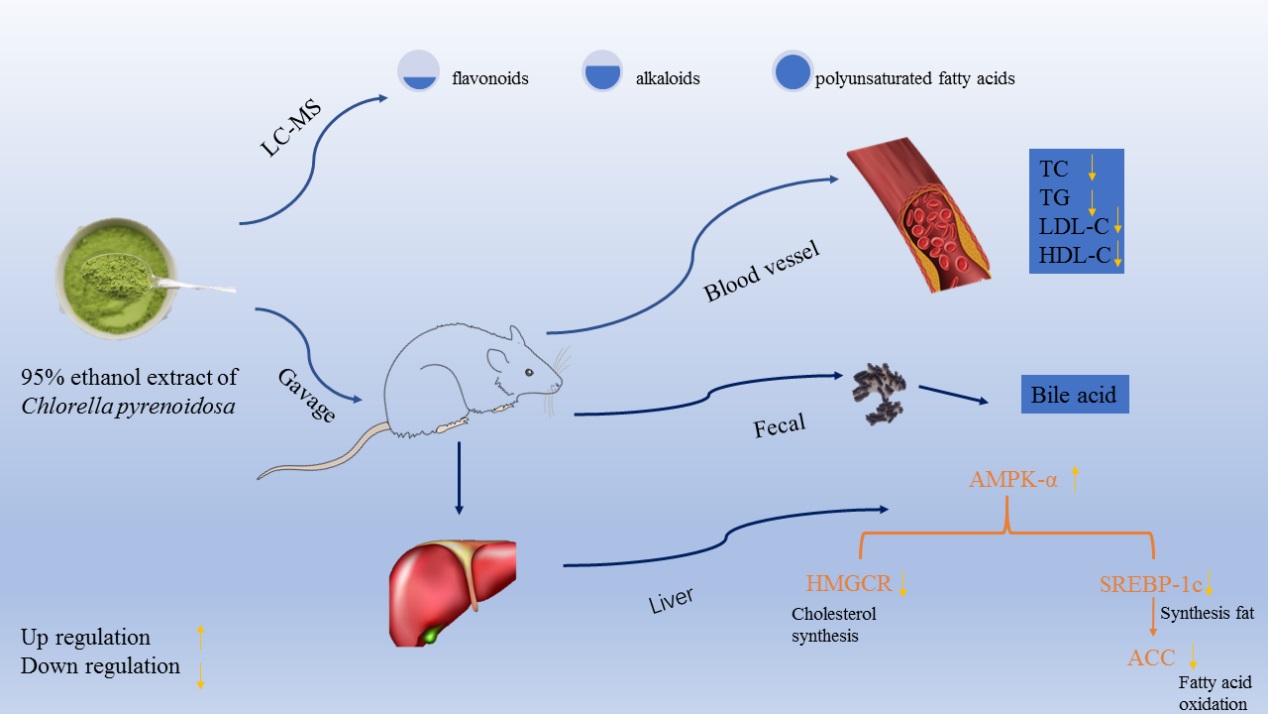


**Figure S2.** The potential molecular mechanisms underlying the hypolipidemic effects of CPE95 in high fat diet induced rats.
